# Unexpected Ecological Resilience in Bornean Orangutans and Implications for Pulp and Paper Plantation Management

**DOI:** 10.1371/journal.pone.0012813

**Published:** 2010-09-22

**Authors:** Erik Meijaard, Guillaume Albar, Nardiyono, Yaya Rayadin, Marc Ancrenaz, Stephanie Spehar

**Affiliations:** 1 People and Nature Consulting International, Jakarta, Indonesia; 2 School of Archaeology and Anthropology, Australian National University, Canberra, Australian Capital Territory, Australia; 3 Société d'Ornithologie de Polynésie, Taravao, Tahiti, French Polynesia; 4 The Nature Conservancy-Indonesia Program, Balikpapan, East Kalimantan, Indonesia; 5 Graduate School of Environmental Science, Hokkaido University, Sapporo, Japan; 6 Forestry Faculty, Mulawarman University, Samarinda, East Kalimantan, Indonesia; 7 Hutan, Sandakan, Malaysia; 8 Department of Religious Studies and Anthropology, University of Wisconsin, Oshkosh, Wisconsin, United States of America; Smithsonian's National Zoological Park, United States of America

## Abstract

Ecological studies of orangutans have almost exclusively focused on populations living in primary or selectively logged rainforest. The response of orangutans to severe habitat degradation remains therefore poorly understood. Most experts assume that viable populations cannot survive outside undisturbed or slightly disturbed forests. This is a concern because nearly 75% of all orangutans live outside protected areas, where degradation of natural forests is likely to occur, or where these are replaced by planted forests. To improve our understanding of orangutan survival in highly altered forest habitats, we conducted population density surveys in two pulp and paper plantation concessions in East Kalimantan, Indonesia. These plantations consist of areas planted with fast-growing exotics intermixed with stands of highly degraded forests and scrublands. Our rapid surveys indicate unexpectedly high orangutan densities in plantation landscapes dominated by *Acacia* spp., although it remains unclear whether such landscapes can maintain long-term viable populations. These findings indicate the need to better understand how plantation-dominated landscapes can potentially be incorporated into orangutan conservation planning. Although we emphasize that plantations have less value for overall biodiversity conservation than natural forests, they could potentially boost the chances of orangutan survival. Our findings are based on a relatively short study and various methodological issues need to be addressed, but they suggest that orangutans may be more ecologically flexible than previously thought.

## Introduction

Over the last few decades, the world's primary and modified natural forests have been much reduced, especially in the tropics. At the same time, however, this loss has to some extent been compensated by an increase in the area of semi-natural forests and forest plantations [1]. These changes are generally thought to negatively impact forest wildlife [2], [3], [4], but this is not always the case [5], [6]. If these altered forests can provide suitable habitat for many forest species, their role in forest wildlife conservation needs to be reconsidered. Species may be able to survive in the matrix created by timber plantations and agro-forestry areas, especially in areas where the landscape also includes large natural forest fragments [7], [8], [9], [10], [11]. The survival of individual species in such anthropogenically-altered forests will depend on their ability to cope with the ecological changes associated with deforestation and plantation development, and the extent to which other threats such as hunting and fire are controlled [12]. As expanding human influence is likely to further fragment and degrade once contiguous forests in tropical areas, an improved understanding of the role these altered landscapes can play in conservation is a high priority.

Southeast Asia experiences some of the highest levels of forest conversion in the world, including deforestation, forest degradation and fragmentation [13] and clearing for rapid expansion of the plantation sector [14]. The high levels of species diversity and endemism in the region's natural forests, combined with these high rates of habitat conversion, makes Southeast Asia a high priority for global biodiversity conservation [14], [15]. These same factors also make them an important testing ground for evaluating the role of degraded and planted forests in biodiversity conservation. If selectively logged and planted forests retain high conservation values (as some studies suggest, at least for selectively logged forests [16]), this calls for the integration of these forests into broader conservation planning. Presently, however, the focus of the conservation movement is mostly on remaining contiguous patches of primary or lightly degraded forest [17], and few studies have addressed the potential conservation role of surrounding matrix habitats, especially planted forests. In this paper, we present the results of a survey carried out in two plantation areas in East Kalimantan. These primarily consist of fast-growing *Acacia mangium* and *Eucalyptus pelita*, grown for the production of pulp and paper. We use these results to assess the potential value of human-dominated matrix habitats, especially plantation areas, for the conservation of the Bornean orangutan (*Pongo pygmaeus*), a globally recognized conservation icon.

Orangutans (*Pongo* spp.) are confined to parts of Malaysian and Indonesian Borneo and the north of Sumatra, Indonesia; both species (*Pongo pygmaeus*, and the Sumatran orangutan, *P. abelii*) are threatened with near-future extinction in the wild [18]. Deforestation and forest degradation negatively affect orangutans [19], [20], [21], although exceptions have been noted [22], [23]. Predictions of further population declines for orangutans are based on estimates of the amount of orangutan habitat that has disappeared and the assumption that these trends will continue in the future. The general assumption is that orangutans lack the resilience and adaptability to cope with deforestation or severe forest degradation. For over two decades, however, chance observations indicate that the Bornean species also survives in degraded forests and even monocultural plantations of exotic tree species (YR, EM and MA, pers. obs.). Such observations have largely been ignored by the scientific community. This suggests that potentially viable orangutan populations are not getting the conservation attention they require. Objective assessments of species' resilience and adaptability are of crucial importance to develop management recommendations that maximize the long-term survival chances of endangered wildlife, especially when rates of forest conversion are high and human-altered landscapes dominate their range. The orangutan appears to be an especially suitable study object, because of its internationally recognized conservation appeal, but also the strongly emotive aspects of orangutan conservation that can potentially cloud objective conservation assessments [24].

**Figure 1:**
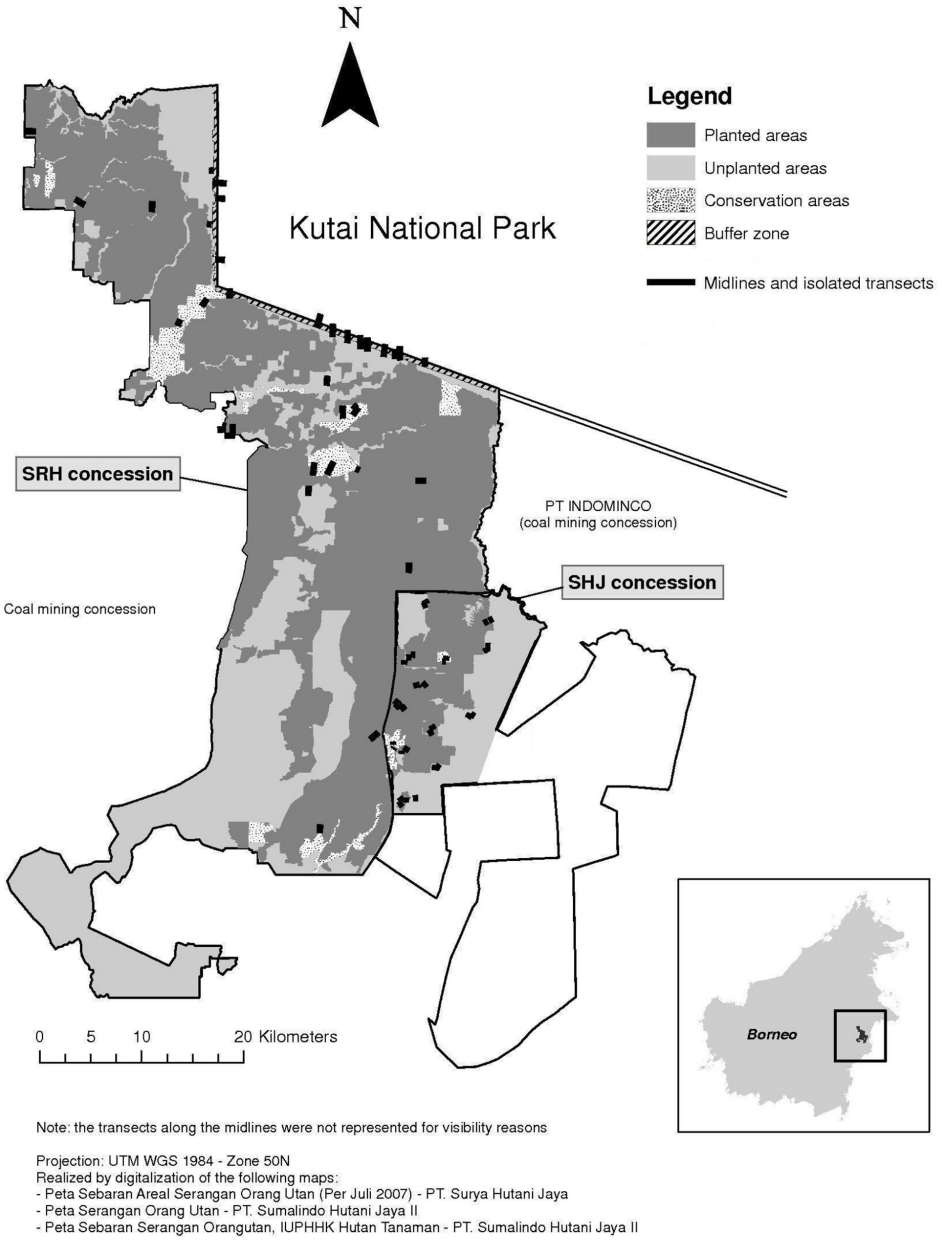
Study area. Map of the study area showing the two plantation areas and the adjacent Kutai National Park and its location in the east of the island of Borneo. Black lines indicate the location of the midlines in the SRH planted, unplanted and conservation areas, and non-randomly placed transects in other parts of the two plantations as well as the buffer zone with Kutai National Park.

We estimated population densities of Bornean orangutans in an area that was largely deforested during the 1980s and 1990s, with forest integrity further impacted by major fire events in 1997 and 2004 [25], [26], [27]. The 199,000 ha Kutai National Park (KNP) forms the conservation core of this region (Fig. 1). This park has been poorly managed and illegal logging, boundary changes and fires [28] have only left ca. 23% of the park forested [29]. Surrounding KNP is a patchwork of plantations for pulp and paper, as well as palm oil production, coal and nickel mines, community lands, and an inactive timber concession. Prior to this widespread alteration and loss of primary forest, this region was thought to contain significant orangutan populations [30], [31]. Afterwards, it seemed highly unlikely that this environmentally degraded area would maintain viable orangutan populations, and the populations that had initially been mapped in this area were removed from the more recent species distribution range maps [32]. It therefore came as a surprise when, in 2007, the timber plantation companies working in the area reported frequent encounters with orangutans and significant damage caused by these animals to stands of young acacias, with orangutans apparently first starting to feed on acacia in 1996 [33]. The authors of this paper investigated the situation to recommend management actions to maximize chances of survival of this remnant orangutan population as well as mitigating the damage caused to the acacia trees. The study specifically targeted two plantation concessions, with a focus on determining the relative importance of different vegetation types in the concessions for orangutans, as well as the potential of certain areas to serve as corridors between remnants stands of natural forest.

## Results

We conducted 144 orangutan nest transect surveys, with a total length of 89.62 km, in two pulp and paper concession areas (SRH and SHJ) in East Kalimantan, Indonesian Borneo. The two concessions are stratified into planted areas, unplanted areas, buffer zones, and conservation areas. Within the planted areas, only those younger than five years and older than one year were surveyed (see Methodology). A total of 937 nests were recorded during these surveys (Table 1). We only calculated nest densities in the SRH concession, because in SHJ and in the buffer zone transects were not randomly placed, and nest data therefore provide only reliable presence rather than density information. Even so, the buffer zone and all parts of the SHJ concession, including the planted areas, were obviously used by orangutans with 594 nests along 37.5 km of transect indicating a high number of nests in that area (see kilometric index in Table 1).

**Table 1:** Estimates of orangutan density and population size in different vegetation types, kilometric indices for areas where no density estimate could be obtained, and total population estimate for the two concession areas.

Type of habitat	Number of nests	Survey effort (km)	Kilometric index (nests/km)	Mean nest decay rate (lower– upper estimate)	Mean density of orangutans (ind/km^2^) (lower– upper estimate)	Area (km^2^)	Mean number of orangutans (lower– upper estimate)
SRH planted areas	167	31.5	5.3	123 (102–144)	1.45 (1.24–1.75)	314.2	456 (390–550)
SRH buffer zone	272	22.65	12.0			21.7	
SRH conservation areas	92	10.03	9.2	153 (60–243)	1.76 (1.11–4.5)	68.0	120 (75–306)
SRH unplanted areas	84	10.64	7.8	222 (119–323)	1.34 (0.92–2.5)	585.6	785 (539–1464)
SHJ planted areas	101	7.32	13.8			72.3	
SHJ conservation areas	38	1.48	25.7			4.45	
SHJ unplanted areas	183	6	30.5			118.5	
**POPULATION ESTIMATE**							**1361 (1004–2320)**

We estimated the population size in the SRH concession by assuming that the orangutan density in the unsurveyed planted areas (those areas >5 years and <1 year old) was 0. This reduces the planted area for which we do have density estimates from the total of 440 km^2^ to 314 km^2^ (Table 1). The resulting orangutan density estimate for the planted area is 1.45 ind/km^2^, which is similar to the density estimates from the conservation areas and unplanted areas (Table 1). Assuming that our survey results are representative of the remaining parts of the concession, this approach provides us with a minimum population estimate of 1,361 animals in the SRH concession (Table 1). The orangutan population of the total plantation area might well be higher than 1,361 animals because our estimates do not include the populations in SHJ and SRH buffer zone, for which no densities could be obtained but where a high number of nests was encountered (Table 1). An ultra-conservative density estimate, using extreme values for key parameters used to convert nest densities into orangutan densities (the rate of nest building *r* and the nest decay time *t*) is 0.44 ind/km^2^. This extrapolates to 577 orangutans in planted, unplanted and conservation areas of SRH, a number which we consider the absolute minimum for the two plantation concessions. The two plantation areas thus contain a population of at least 1% of the total Bornean orangutan population [18], but possibly as high as 3 or 4%.

## Discussion

We emphasize that the data presented here provide only a preliminary picture of orangutan use of acacia and eucalyptus plantation landscapes, and we would like to address several potential sources of methodological bias before assessing the conservation implications of our findings. First, this study assumes that the nest building rates are the same for orangutans in plantations and in natural forests. Feeding and resting patterns of orangutans in plantations might, however, differ compared to animals in natural forests. There are indications that orangutans rest more when there is less fruit available [34]. The frequency of nest building also varies based on fruit availability, especially for day nests [35]. This is thought to be linked to the time spent feeding on fruit versus a lower-quality food source, inner bark [35], and the availability of fruit varies significantly between plantations and natural forest fragments. Secondly, the scarcity of suitable nest trees in plantations predominantly consisting of acacia trees may also increase the frequency of nest re-use. Until better data become available about nest building rates in plantations we cannot be sure whether we overestimate or underestimate population densities by using a nest building parameter (*r*) obtained from populations living in natural forest.

Despite these limitations, the key finding of this study is that orangutans use acacia plantation landscapes. This does not mean that plantations have the same conservation value as natural forests, but, at least for orangutans, they have some value that in the past has not been sufficiently recognized. It is still too early to know whether these populations are transient individuals in search of new forest habitat, or whether this area is part of a recolonization process from nearby over-degraded forests. The long-term viability of these populations also requires further study. It is almost certain that their survival depends not just on plantations but on connectivity to resources available elsewhere in the landscape, including the adjacent national park, and we emphasize that plantations cannot be viewed as stand-alone “conservation solutions” but only as a part of a larger mixed landscape upon which orangutans rely. Still, the fact that orangutans are found in landscapes that have been highly degraded for nearly 20 years has important conservation implications. With more than 1.3 million ha of such plantations presently under development in West and East Kalimantan [36], at least some of which fall within the orangutan's potential distribution, a significant number of orangutans could find themselves in similar situations as those reported in the present study.

Although it is clear that orangutans are using these degraded habitats, it remains unclear how plantations of *A. mangium* and surrounding vegetation scrub can support orangutan populations. Orangutans normally rely on a diet of fruits, flowers, and leaves, and to a lesser extent bark, invertebrates and other food items [34]. But the intake of each of these categories varies considerably throughout the year and between different sites [34], [37]. Some preliminary botanical surveys in the plantations suggest that few trees remained that would provide edible fruit or flowers. This implies that orangutans in the acacia plantations obtain most of their energy from what would normally be considered as “fallback foods” [38]: bark and leaves. Our direct observation of an orangutan feeding on acacia as well as numerous records of bark stripping by orangutans in *A. mangium* plantations suggest that the cambium and likely sap provide an important food source for orangutans when they are feeding in the planted areas. To what extent orangutans rely on and obtain additional food items remains unclear. Furthermore, interviews with company staff and preliminary data from other local studies suggest that orangutans actively move through the landscape, possibly more than they would in their natural forest habitat. We do not know to what extent the adjacent national park provides a regular source of food or habitat in addition to what the animals find in the plantation area. These questions, among other, will be addressed in a planned follow up study.

The conservation implications of these findings are important, suggesting that we must make efforts to enhance the orangutan's chances of survival in plantation forests and the surrounding matrix habitats. Both concession areas that were the subject of this study were made available for planting after the natural forest had been largely removed by two decades of unsustainable timber harvest, illegal logging and fire. The concessions were legally granted under the correct assumptions that the forest in the area was gone or severely degraded. What was overlooked by government organizations and conservation groups is that the area retained a significant population of orangutans, and possibly other protected species [39]. Orangutans are protected in Indonesia and it is illegal to kill, move, or trade orangutans [40]. There are, however, no legal prescriptions on how to retain, improve, or manage orangutan habitat. From an objective point of view, this creates a dilemma for plantation companies. Orangutans cause significant damage to acacia and companies report that 5–10% of each planting cohort in the study concessions is killed by bark stripping (as observed by us on maps of damaged plantation areas and subsequent field checks). In the past, both companies have asked for help from the authorities to translocate orangutans out of the plantations, but this is logistically difficult, impractical, and expensive [41]. Instead we recommend solutions that resolve orangutan management issues *in situ* by trying to reconcile ecological needs of the species with the economic development goals of plantations, for example by increasing the size and interconnectedness of conservation areas and adjacent forested habitat. The companies are presently implementing some of our preliminary recommendations, including a revised macro-level spatial plan for increasing the size of conservation areas and their interconnectedness. Ongoing and future research focuses on more detailed management recommendations to retain viable populations of orangutans and other wildlife in the plantations and the surrounding matrix habitat.

Tropical forests are likely to undergo further degradation and fragmentation. The present example of orangutans in pulp and paper plantation emphasizes the urgent need for the conservation science community to focus on the potential of multifunctional forest and plantation landscapes to provide at least some resources to endangered wildlife. The majority of orangutans occur outside national parks [42], and unfortunately many of their habitats will be used for commercial timber extraction, and converted to timber plantations and other plantations such as oil palm (*Elaeis guineensis*). Although Indonesia has announced a two-year moratorium on new forest clearance, which may represent a trend towards less forest conversion in the future, all concessions granted before this agreement will likely be allowed to proceed with extractive activities. We need to know whether and which species use, and can survive in, degraded habitats that make up the matrix outside forest reserves and how their survival could be supported through better management of the entire landscape. Focusing mainly on the management of contiguous, intact reserves and ignoring the conservation opportunities of such multifunctional landscapes would be a lost opportunity for conservation.

## Materials and Methods

Surveys were conducted in February, June and August 2008 in two plantation concessions located in East Kalimantan, Indonesia: Surya Hutani Jaya (SRH), which is approximately 165,000 ha of which 98,000 ha are planted (ca. 90% *A. mangium* and 10% *Eucalyptus pelita*); and Sumalindo Hutani Jaya (SHJ), which is approximately 73,000 ha of which 11,000 ha are planted with *A. mangium* (Fig. 1). Both concessions have followed Indonesian legal requirements to set aside 10% of their concession as conservation area, and a further 20% for local use and indigenous species, but the vegetation in these conservation areas consists of highly degraded forest at best. In the SHJ concession, only the western part was surveyed because of logistical constraints (hereafter, “SHJ concession” will refer to the western part of the SHJ concession).

Orangutans are generally surveyed by counting orangutan sleeping platforms (or ‘nests’) along line-transects as an indirect method to estimate orangutan densities [23], [43], [44], [45]. We employed this methodology to determine density variation among different habitat types. Based on vegetation maps provided by the companies, we laid transect systems in four different habitat types: planted areas, unplanted protected areas (referred to as “conservation areas”), unplanted unprotected areas (referred to as “unplanted areas”), and the buffer zone between the SRH concession and the adjacent KNP. The latter category was set apart due to its particular status and ecological importance (connection between the SRH concession and KNP).

We used three survey methods to rapidly produce an overall picture of orangutan use of the different vegetation types in the survey areas. In the SRH concession, we placed seven or eight, 500 m transects perpendicularly to 1 km-long midlines, with the stipulation that two transects on the same side of a midline were at least 100 m from each other to avoid double-counting a nest in two transects; midlines were randomly placed in the targeted types of habitat (see Table 1). The position of each transect along a midline was randomly determined using a stopwatch [46]. This method was employed in the planted and in most of the unplanted areas of the SRH concession. Secondly, rapid surveys were used to check whether the situation documented in SRH was similar in SHJ. The shape and size of the conservation forest fragments often made it impossible to randomly place 500 m transects without exiting the fragment. Because of this constraint, we only conducted reconnaissance surveys along non-random transects, which allowed us to determine kilometric indices (nests/km) but not nest densities. These surveys were done by placing isolated 500 m transects in the targeted types of habitat. Finally, in addition to the previous two approaches, we non-randomly placed some 1,500 m transects in the buffer zone between the SRH concession and KNP (see Fig. 1) to survey the entire width of the buffer zone.

The total transect length in a given habitat type was not proportional to the respective areas of the habitat types. A bias in survey effort towards the planted areas and the buffer zone was introduced to generate as much information as possible for direct translation into management recommendations for the plantation companies and national park authority. Among the planted areas, only those planted between mid-2003 and the end of 2006 were surveyed and taken into account in the calculations, because preliminary surveys [33] suggested that orangutans did not use trees younger than 1 year or older than 5 years for nesting sites (the latter possibly because there is a great deal of pre-harvest human activity).

The areas of the different habitat types were calculated with Geographic Information System (GIS) software, except the areas planted between mid-2003 and the end of 2006 which were directly obtained from plantation company maps.

Data were collected by teams of at least two experienced surveyors [23], [47], [48]. Each transect was censused twice, in opposite directions, to minimize the chance that nests were missed [43]. For each nest, the tree species was noted, and the perpendicular distance between the point vertically below the nest and the transect was measured using a triple decametre. Nests that were more than 60 m from the transects were not taken into account for the density calculations due to concerns about accuracy of the estimation of perpendicular distances [49], [50]. Previous checks ensured that tree names were consistent between teams.

The data were analyzed independently for each concession and each type of habitat. Following van Schaik et al. (1995), the equation below was used to calculate the density of orangutan nests (*D_N_*):
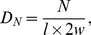
where *N* is the number of nests observed along the transect, *l* the length of the transect (m) and *w* the estimated width of the strip effectively sampled along the transect (m).

The software Distance 5.0 Release 2 [51] was used to estimate *w*. For each set of data, several mathematical models were fit, and the one which best matched the data was chosen, based on the lowest value for Akaike's Information Criterion (AIC).

Subsequently, *D_N_* was used to calculate the density of orangutans (*D_OU_*) in each region as follows:
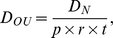
where *p* is the proportion of nest builders in the population, *r* is the rate of nest building (nests/day/individual) and *t* is the nest decay time, or the amount of time a nest is recognizable before it decays (in days). We used *p* = 0.9, based on data from several studies [45] that indicate that this parameter is consistent between Sumatran and Bornean populations. We used a Borneo-specific *r* = 1.08, following [43], [52], [53] which is the average of the only two published nest production rate values for Borneo: 1.01 at Kinabatangan, Malaysia [53] and 1.16 at Gunung Palung, West Kalimantan [43].

The second major constraint in this study is the estimation of *t*, which we know to be subject to high spatial and temporal variation [48]. For all planted areas of the two concessions, we used a decay rate of 123±21 days (n = 68), following a nest decay rate study conducted in the areas of the SHJ concession planted with *Acacia mangium* in 2007–2008 [54]. Knowing that nest decay rates are strongly influenced by tree species and climate conditions [48], [53], we used decay rates from another site in eastern Borneo with similar heavily disturbed forests and rainfall patterns to calculate orangutan densities in all the other habitat types [53]. Ancrenaz et al. [53] found that the nest decay time can vary considerably between tree species; in particular, nests built with the hard *Eusideroxylon* sp. wood have very long decay rates (476±140 days), and the abundance of this tree has to be taken into account when calculating the nest decay rates in a given region. Ancrenaz et al. [53] grouped all the other tree species, evaluating the mean nest decay rate to be 153±93 days. Thus, for each of the 5 unplanted regions surveyed in this study, the following formula was used to calculate a specific nest decay rate:
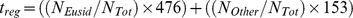
with *t_reg_* the nest decay rate specific to a given region, *N_Eusid_* the number of nests made in *Eusideroxylon* sp. trees, *N_Other_* the number of nests made in other tree species and *N_Tot_* the total number of nests found in the region.

To obtain a minimum estimate of the orangutan population in the study area, we also calculated a population density using the most extreme available values for key parameters: a nest decay rate (*t*) of 628 days [48] and a nest building rate (*r*) of 2 nests/day/individual, rather than the average Borneo-specific value of 1.08 [43], [52], [53].

Our approach thus uses two sets of estimates, one which is conservative and one which is ultra-conservative. Only density estimates from the planted areas are used even though orangutans make extensive use of the conservation areas. The estimate derived from extreme decay rate values provides what we believe to be an absolute minimum population size for the entire plantation area.

## References

[1] FAO (2006). Global forest resources assessment 2005..

[2] Myers N (1996). The worlds forests - problems and potentials.. Env Cons.

[3] Lawton JH, Bignell DE, Bolton B, Bloemers GF, Eggleton P (1998). Biodiversity inventories, indicator taxa and effects of habitat modification in tropical forest.. Nature.

[4] Brook BW, Sodhi NS, Ng PKL (2003). Catastrophic extinctions follow deforestation in Singapore.. Nature.

[5] Brockerhoff EG, Jactel H, Parrotta JA, Quine CP, Sayer J (2008). Plantation forests and biodiversity: oxymoron or opportunity?. Biodivers Conserv.

[6] Chazdon RL, Peres CA, Dent D, Sheil D, Lugo AE (2009). The potential for species conservation in tropical secondary forests.. Conserv Biol.

[7] Chazdon RL (2008). Beyond deforestation: Restoring forests and ecosystem services on degraded lands.. Science.

[8] Henle K, Davies KF, Kleyer M, Margules C, Settele J (2004). Predictors of species sensitivity to fragmentation.. Biodivers Conserv.

[9] Wright SJ, Muller-Landau HC (2006). The uncertain future of tropical forest species.. Biotropica.

[10] Wilson K, Meijaard E, Drummond S, Grantham H, Boitani L (in press). Conserving biodiversity in production landscapes.. Ecol Appl.

[11] Franklin J, Lindenmeyer DB (2009). Importance of matrix habitats in maintaining biological diversity.. Proc Nat Acad Sc USA.

[12] Meijaard E, Sheil D, Nasi R, Augeri D, Rosenbaum B (2005). Life after logging: reconciling wildlife conservation and production forestry in Indonesian Borneo.

[13] Stibig H-J, Stolle F, Dennis R, Feldkötter C (2007). Forest Cover Change in Southeast Asia..

[14] Koh LP (2007). Impending disaster or sliver of hope for Southeast Asian forests? The devil may lie in the details.. Biodivers Conserv.

[15] Sodhi NS, Koh LP, Brook BW, Ng PKL (2004). Southeast Asian biodiversity: An impending disaster.. Tr Ecol Evol.

[16] Ancrenaz M, Ambu L, Sunjoto I, Ahmad E, Manokaran K (2010). Recent surveys in the forests of Ulu Segama Malua, Sabah, Malaysia, show that orang-utans (*P. p. morio*) can be maintained in slightly logged forests.. PLoSONE.

[17] Koh LP, Ghazoul J, Butler RA, Laurance WF, Sodhi NS (2009). Wash and spin cycle threats to tropical biodiversity.. Biotropica.

[18] Wich SA, Meijaard E, Marshall AJ, Husson S, Ancrenaz M (2008). Distribution and conservation status of the orang-utan (*Pongo* spp.) on Borneo and Sumatra: how many remain?. Oryx.

[19] Rijksen HD, Meijaard E (1999). Our vanishing relative. The status of wild orang-utans at the close of the twentieth century.

[20] Morrogh-Bernard H, Husson S, Page SE, Rieley JO (2003). Population status of the Bornean orang-utan (*Pongo pygmaeus*) in the Sebangau peat swamp forest, Central Kalimantan, Indonesia.. Biol Cons.

[21] Felton AM, Engstrom LM, Felton A, Knott CD (2003). Orangutan population density, forest structure and fruit availability in hand-logged and unlogged peat swamp forests in West Kalimantan, Indonesia.. Biol Cons.

[22] Knop E, Ward PI, Wich SA (2004). A comparison of orang-utan density in a logged and unlogged forest on Sumatra.. Biol Cons.

[23] Marshall AJ, Nardiyono, Engstrom LM, Pamungkas B, Palapa J (2006). The blowgun is mightier than the chainsaw in determining population density of Bornean orangutans (*Pongo pygmaeus morio*) in the forests of East Kalimantan.. Biol Cons.

[24] Sheil D, Meijaard E (in press). Purity and prejudice: deluding ourselves about biodiversity conservation.. Biotropica.

[25] Dennis RA, Colfer CP (2006). Impacts of land use and fire on the loss and degradation of lowland forest in 1983–2000 in East Kutai District, East Kalimantan, Indonesia.. Singap J Trop Geogr.

[26] Malingreau JP, Stephens G, Fellows L (1985). Remote sensing of forest fires: Kalimantan and North Borneo in 1982–83.. Ambio.

[27] Siegert F, Ruecker G, Hinrichs A, Hoffmann AA (2001). Increased damage from fires in logged forests during droughts caused by El Nino.. Nature.

[28] MacKinnon K, Irving A, Bachruddin MA (1994). A last chance for Kutai National Park-local industry support for conservation.. Oryx.

[29] Hartman P, Kitchener DJ, Adler R, Crevello S, Fong C (2010). Orangutan Conservation Services Program..

[30] Doi T (1986). Present status of the large mammals in the Kutai National Park, after a large scale fire in East-Kalimantan, Indonesia.

[31] Witkamp H (1932). Het voorkomen van enige diersoorten in het landschap Koetai.. Trop Natuur.

[32] Singleton I, Wich SA, Husson S, Atmoko SU, Leighton M (2004). Orangutan Population and Habitat Viability Assessment: Final Report.

[33] Rayadin Y (2007). Laporan hasil monitoring keberadaan populasi orangutan di kawasan Hutan Tanaman Industri PT..

[34] Morrogh-Bernard H, Husson SJ, Knott CD, Wich SA, van Schaik CP, Wich SA, Atmoko SU, Setia TM, van Schaik CP (2009). Orangutan activity budgets and diet. A comparison between species, populations and habitats.. Orangutans Geographic variation in behavioral ecology and conservation.

[35] Prasetyo D, Ancrenaz M, Morrogh-Bernard HC, Atmoko SUW, S.A., van Schaik CP, Wich SA, Atmoko SU, Setia TM, van Schaik CP (2009). Nest building in orangutans.. Orangutans Geographic variation in behavioral ecology and conservation.

[36] Barr C (2008). Indonesia's Pulp & Paper Industry: Overview of Risks and Opportunities.. http://www.environmentalpaper.org/documents/Chris%20Barr%20-%20CIFOR.pdf.

[37] Russon AE, Wich SA, Ancrenaz M, Kanamori T, Knott CD, Wich S, Atmoko SU, Setia TM, van Schaik CP (2009). Geographic variation in orangutan diets.. Orangutans Geographic variation in behavioral ecology and conservation.

[38] Marshall AJ, Wrangham R (2007). Evolutionary consequences of fallback foods.. Int J Primat.

[39] McShea WJ, Stewart C, Peterson L, Erb P, Stuebing R (2009). The importance of secondary forest blocks for terrestrial mammals within an Acacia/secondary forest matrix in Sarawak, Malaysia.. Biol Conserv.

[40] Ministry of Forestry (1990).

[41] Andau PM, Hiong LK, Sale JB (1994). Translocation of pocketed orang-utans in Sabah.. Oryx.

[42] Meijaard E, Wich S (2007). Putting orang-utan population trends into perspective.. Curr Biol.

[43] Johnson AE, Knott CD, Pamungkas B, Pasaribu M, Marshall AJ (2005). A survey of the orangutan (*Pongo pygmaeus wurmbii*) population in and around Gunung Palung National Park, West Kalimantan, Indonesia based on nest counts.. Biol Cons.

[44] Marshall AJ, Meijaard E (2009). Orangutan nest surveys: the devil is in the details.. Oryx.

[45] Schaik van CP, Azwar, Priatna D, Nadler RD, Galdikas BFM, Sheeran LK, Rosen N (1995). Population estimates and habitat preferences of orang-utans based on line transects of nests.. The neglected ape.

[46] Fulton M (1996). The digital stopwatch as a source of random numbers.. Bull Ecol Soc Amer.

[47] Marshall AJ, Salas LA, Stephens S, Nardiyono, Engstrom L (2007). Use of limestone karst forests by Bornean orangutans (*Pongo pygmaeus morio*) in the Sangkulirang Peninsula, East Kalimantan, Indonesia.. Am J Primatol.

[48] Mathewson PD, Spehar SN, Meijaard E, Nardiyono, Purnomo (2008). Evaluating orangutan census techniques using nest decay rates: Implications for population estimates.. Ecol Appl.

[49] Buckland ST, Anderson DR, Burnham KP, Laake JL (1993). Distance Sampling: Estimating Abundance of Biological Populations.

[50] Buckland ST, Anderson DR, Burnham KP, Laake JL, Borchers DL (2001). Introduction to Distance Sampling.

[51] Thomas L, Laake JL, Strindberg S, Marques FFC, Buckland ST (2006). Distance 5.0. Release 2. Research Unit for Wildlife Population Assessment.. http://www.ruwpa.st-and.ac.uk/distance/.

[52] Ancrenaz M, Gimenez O, Ambu L, Ancrenaz K, Andau P (2005). Aerial surveys give new estimates for orangutans in Sabah, Malaysia.. Plos Biol.

[53] Ancrenaz M, Goossens B, Gimenez O, Sawang A, Lackman-Ancrenaz I (2004). Determination of ape distribution and population size using ground and aerial surveys: A case study with orang-utans in lower Kinabatangan, Sabah, Malaysia.. Anim Cons.

[54] Rayadin Y, Saitoh T (2009). Individual variation in nest size and nest site features of the Bornean orangutans (*Pongo pygmaeus*) (vol 71, pg 393, 2009).. Am J Primatol.

